# Psychosocial stimulation interventions for children with severe acute malnutrition: a systematic review

**DOI:** 10.7189/jogh.07.010405

**Published:** 2017-06

**Authors:** Allison I Daniel, Robert H Bandsma, Lyubov Lytvyn, Wieger P Voskuijl, Isabel Potani, Meta van den Heuvel

**Affiliations:** 1 Centre for Global Child Health, Hospital for Sick Children, Toronto, Canada; 2 Department of Nutritional Sciences, University of Toronto Faculty of Medicine, Toronto, Canada; 3 Division of Gastroenterology, Hepatology and Nutrition, Hospital for Sick Children, Toronto, Canada; 4 Child Health Evaluative Sciences, Hospital for Sick Children, Toronto, Canada; 5 Department of Paediatrics and Child Health, College of Medicine, University of Malawi, Blantyre, Malawi; 6 Global Child Health Group, Emma Children’s Hospital, Academic Medical Centre, University of Amsterdam, Amsterdam, The Netherlands; 7 Nutritional Rehabilitation Unit, Queen Elizabeth Central Hospital, Blantyre, Malawi; 8 Division of Paediatric Medicine, Hospital for Sick Children, Toronto, Canada; 9 Department of Paediatrics, University of Toronto Faculty of Medicine, Toronto, Canada

## Abstract

**Background:**

The WHO *Guidelines for the inpatient treatment of severely malnourished children* include a recommendation to provide sensory stimulation or play therapy for children with severe acute malnutrition (SAM). This systematic review was performed to synthesize evidence around this recommendation. Specifically, the objective was to answer the question: “In children with severe acute malnutrition, does psychosocial stimulation improve child developmental, nutritional, or other outcomes?”

**Methods:**

A review protocol was registered on the International Prospective Register of Systematic Reviews (PROSPERO 2016: CRD42016036403). MEDLINE, Embase, CINAHL, and PsycINFO were searched with terms related to SAM and psychosocial stimulation. Studies were selected if they applied a stimulation intervention in children with SAM and child developmental and nutritional outcomes were assessed. Findings were presented within a narrative synthesis and a summary of findings table. Quality of the evidence was evaluated using the Cochrane risk of bias tool and the Grading of Recommendations Assessment, Development and Evaluation (GRADE) approach.

**Findings:**

Only two studies, both non–randomized controlled trials, met the selection criteria for this review. One was conducted in Jamaica (1975) with a follow–up period of 14 years; the other was done in Bangladesh (2002) with a six–month follow–up. At the individual study level, each of the included studies demonstrated significant differences in child development outcomes between intervention and control groups. Only the study conducted in Bangladesh demonstrated a clinically significant increase in weight–for–age z–scores in the intervention group compared to the control group.

**Conclusions:**

The evidence supporting the recommendation of psychosocial stimulation for children with SAM is not only sparse, but also of very low quality across important outcomes. High–quality trials are needed to determine the effects of psychosocial stimulation interventions on outcomes in children with SAM.

Malnutrition, particularly in the first 1000 days of life, is known to be associated with serious outcomes including increased vulnerability to infection and disease, compromised development, as well as mortality [1–3]. In this same period of time, evidence suggests that inadequate psychosocial stimulation (ie, physical, sensory, and/or emotional input) inhibits infants from achieving developmental potential [3–5]. Malnutrition combined with psychosocial deprivation can have considerable implications on child development that last throughout life including reduced intellectual capacity, and at a larger scale this can result in reduced societal contribution [5–7]. Because of the importance of early child development for country–level progress, the Sustainable Development Goals (SDG) have now included a focus for children younger than five years to achieve developmental milestones: “By 2030 ensure that all girls and boys have access to quality early childhood development, care and pre–primary education so that they are ready for primary education” (Target 4.2) [8].

Children with severe acute malnutrition (SAM) are at exceptionally high risk of poor growth outcomes and are also thought to be at high risk for motor and cognitive delays, as brain development is further inhibited with increasing severity of malnutrition [3,9,10]. SAM is defined by weight–for–length z–scores (WLZ) or weight–for–height z–scores (WHZ) at least three standard deviations below the median, a mid–upper arm circumference (MUAC) less than 115 mm, and/or nutritionally–induced bilateral pitting edema [11]. Children with WLZ or WHZ and/or MUAC meeting the above criteria are indicative of marasmus or severe wasting, while the presence of bilateral pitting edema is indicative of kwashiorkor [11]. Current guidelines recommend that SAM is treated through Community–Based Management of Acute Malnutrition (CMAM) [12,13]. In critical cases, inpatient treatment is required for children with SAM. This includes children with severe bilateral pitting edema, loss of appetite, or medical complications in addition to SAM [11,13].

Emotional and physical stimulation was first recommended for children with SAM by the World Health Organization (WHO) in the 1999 *Management of severe malnutrition: a manual for physicians and other senior health workers* [14]. The 2003 *Guidelines for the inpatient treatment of severely malnourished children* include ten principles for routine care; one of those is to establish a stimulating environment for children, along with involvement of primary caregivers in caring for and playing with children whenever possible [15]. Specifically, structured play therapy for 15–30 minutes per day is recommended with examples of activities related to language skills and motor development with the use of simple toys [15]. This recommendation was not described or evaluated in the 2013 *Guideline: updates on the management of severe acute malnutrition in infants and children*, but still remains as one of the ten steps of routine inpatient care for children with SAM [15,16]. On the contrary, the CMAM approach does not include recommendations around psychosocial stimulation in children with SAM in the community [12,13].

Psychosocial stimulation in children with SAM has not been evaluated in a rigorous manner in relation to child developmental and nutritional outcomes. Therefore, the primary objective of this systematic review was to synthesize evidence related to the question, “In children with severe acute malnutrition, does psychosocial stimulation improve child developmental, child nutritional, or other child outcomes?”

## METHODS

A review protocol was registered on the International Prospective Register of Systematic Reviews (PROSPERO 2016:CRD42016036403). For reporting of this review, the standard guidelines by the Preferred Reporting Items for Systematic reviews and Meta–Analyses (PRISMA) were followed (see Table S1 in **Online Supplementary Document(Online Supplementary Document)**) [17].

### Search strategy

The search strategy for this review was designed in consultation with a hospital research librarian at the Hospital for Sick Children to ensure a comprehensive search of the literature. The search included terms related to SAM, psychosocial interventions or therapy, and deprivation of psychosocial stimulation, specified in Table S2 in **Online Supplementary Document(Online Supplementary Document)**. There were no language, location, or publication period restrictions applied. Four electronic bibliographic databases were searched up to March 29, 2016: MEDLINE(R) In–Process & Other Non–Indexed Citations (1946 to present), Embase Classic+Embase (1947 to present), CINAHL (1937 to present), and PsycINFO (1806 to present). Reference lists of included studies were also evaluated to identify any potential studies for inclusion.

### Inclusion and exclusion criteria

#### Studies

There were no restrictions on the study time periods or design types eligible for inclusion.

#### Participants

Children (0 to 18 years) with SAM were included; children had to have kwashiorkor (identified by bilateral pitting edema) and/or severe wasting (identified by WLZ or WHZ below –3 SD or MUAC less than 115 mm) [11]. The currently accepted criteria for identification of SAM were developed in 2006 [11,18], thus for studies conducted prior to 2006, anthropometric measures of children were compared to the current measures for SAM by examining previous and recent cut–off values for weight–for–length or –height [18]. If an alternative identification of SAM was used and there was confidence that children enrolled in these studies had anthropometric measures that did not match with the current definition of SAM, these studies were excluded. Studies that focused on children with other types of malnutrition, such as moderate acute malnutrition (MAM), were also excluded.

#### Interventions

Psychosocial stimulation (ie, physical, sensory, and/or emotional input), play therapy, or responsive parenting interventions in any setting (eg, community or hospital–based) were included.

### Controls

Intervention groups were compared to no intervention or alternative intervention groups.

### Primary outcomes

Child developmental (eg, cognitive, language, motor, and social–emotional measures) and nutritional outcomes (eg, anthropometric measures) were specified as the primary outcomes. Anthropometric measures of interest included weight–for–length or –height (ie, indicators of wasting), length– or height–for–age (ie, indicators of stunting), and weight–for–age (ie, indicators of underweight). Body mass index was not defined as an important nutritional outcome for this review because its implications for children and adolescents are indeterminate [19].

### Secondary outcomes

Child quality of life outcomes, morbidities, and mortality were included as secondary outcomes.

### Study selection and data extraction

Two authors (AD and MvdH) independently screened the titles and abstracts, followed by the full texts of potentially eligible studies, for eligibility as per the pre–specified selection criteria. Articles that were not in English (ie, French and Spanish) were translated. Finally, a third author (RB) was consulted to resolve any discrepancies between the two reviewers. Results from the screening process were summarized in a flow diagram as per the PRISMA guidelines [17]. Data from the selected studies were extracted by each of the two reviewers independently, including study information and methods, participant characteristics, intervention properties, and child outcomes.

### Assessment of evidence quality

Each of the two authors independently assessed risk of bias for each study using the Cochrane Handbook for Systematic Reviews of Interventions [20]. Although none of the included studies were randomized–controlled trials, the Cochrane risk of bias tool was deemed suitable because both included studies were experimental and controlled [20]. In addition to the standard six criteria for assessing risk of bias according to the Cochrane Handbook for Systematic Reviews of Interventions, risk of bias from confounding was also examined to account for the fact that participants were not randomized [20]. A risk of bias summary was created using Review Manager 5.3 [21]. The Grading of Recommendations Assessment, Development and Evaluation (GRADE) approach was then used to assess the body of evidence for each outcome [22].

Both studies were non–randomized controlled trials, thus they were considered observational studies in the context of GRADE, and started as low quality of evidence. The quality could have been downgraded for study limitations (risk of bias), inconsistency of results, indirectness of evidence, imprecision, publication bias, or could have been upgraded for large magnitude of effect, confounding, and dose–response gradient [22]. Clinical heterogeneity of outcome measures was qualitatively assessed based on the discretion of authors of this review.

### Analysis

A narrative synthesis was done of all eligible studies. Data were analyzed using Review Manager 5.3 [21]. To summarize findings across studies that included an intervention and comparison group, quantitative analyses were conducted in which mean differences or standardized mean differences with 95% confidence intervals (CI) were calculated for continuous outcomes and risk ratios with 95% CI were calculated for dichotomous outcomes. When outcomes were similar, results across studies were pooled. A summary of findings table was created in GRADEpro 3.6 in which the most important outcomes were included [23]. Results were considered statistically significant if 95% CI did not cross 0. A meta–analysis and subgroup analyses were not conducted, as there were too few studies identified from the search, without similar outcome measures.

## RESULTS

### Study selection

**Figure 1:**
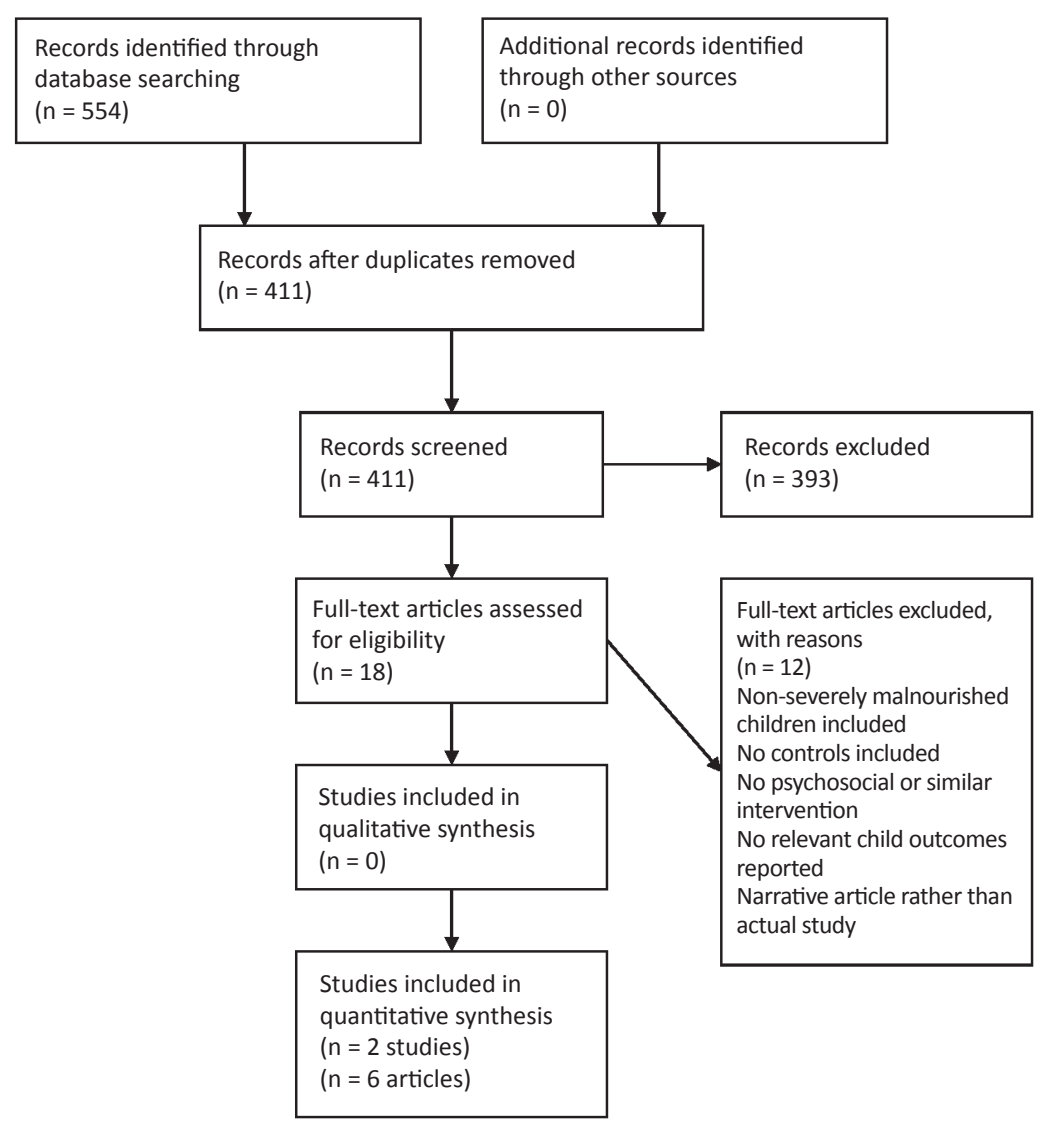
Study flow diagram.

The database search yielded 554 articles, which were narrowed down to 411 articles after duplicates were removed. The results were confined to 18 articles that could potentially meet the inclusion criteria. These remaining articles were assessed in full, with two studies being selected to be included in the systematic review, one of which was published as five separate articles meeting the inclusion criteria [24–29]. No additional studies were identified from the reference lists of included studies. This is summarized as a flow diagram in **Figure 1**. Reasons for excluding studies are listed in Table S3 in **Online Supplementary Document(Online Supplementary Document)**.

### Study characteristics

Two studies met the selection criteria for this review. The first study was conducted in Jamaica by Grantham–McGregor et al. beginning in 1975. Five papers that met the inclusion criteria for this review were published on the same study population. For the purpose of this review, these papers will be referred to as Grantham–McGregor 1980, the first paper published for this study. The second study meeting the inclusion criteria for this review was conducted in Bangladesh by Nahar et al. starting in 2002, and henceforth it will be referred to as Nahar 2009, the year it was published. Grantham–McGregor was also an author of the Nahar 2009 study.

The Grantham–McGregor 1980 study included children with marasmus (identified by authors as having weight below 60% of expected weight for age), marasmic–kwashiorkor (weight below 60% of expected weight for age with edema), or kwashiorkor (weights below 80% of expected weight for age with edema) in the intervention and control groups receiving standard inpatient nutritional care per hospital guidelines, although it is unclear whether these guidelines align with WHO guidelines for SAM treatment. This study also included a third comparison group of non–malnourished children who did not participate in any type of intervention program or treatment.

The Nahar 2009 study compared two malnourished groups of children, including children with marasmus (identified by authors as having weight–for–age below 50% or weight–for–length below 70% of expected values) and kwashiorkor (children with edema), or a combination of the two. Children in both groups received inpatient nutritional care according to the 1999 WHO guidelines for treatment of SAM [14]. Both groups were followed up at seven hospital visits over six months after receiving inpatient care, in which health and nutrition education was done and micronutrient supplements were provided.

The two studies used a similar type of psychosocial intervention with a focus on activities that would stimulate a child’s development including the involvement of mothers to play and talk with their children. In both studies, the intervention started in the hospital and was continued at home. This included one hour per week for two years and one hour every two weeks for the third year after hospital discharge in the McGregor 1980 study, and 18 supervised play sessions (7 play sessions in the hospital and 11 sessions at home) within six months of discharge in the Nahar 2009 study.

The Grantham–McGregor 1980 study used different developmental and IQ tests that were not developed or standardized in Jamaica. The Nahar 2009 study used the Bayley Scales of Infant Development (BSID–II) to assess psychomotor development. The BSID–II is not standardized in Bangladesh, however a strong interobserver reliability of r = 0.99 (*P* < 0.001) was reported. The main characteristics of the two studies, including the developmental assessment tools and the anthropometric measures used, are further described in **Table 1**.

**Table 1:** Study characteristics and primary outcomes

Reference	Design, sample size, mean age (months, SD)	Follow–up	Developmental outcomes (mean, SD)*			Nutritional outcomes*			
			**Cognitive**	**Language**	**Motor**	**Weight expected for height (%, SD)**	**Height expected for age (%, SD)**	**Height for age (mean z–scores, SD)**	**Weight for age (mean z–scores, SD)**
Grantham–McGregor 1980 [27]	Quasi–experimental study, Jamaica; Enrolment: 1975–1977; INT n = *21*, 12.7 ± 3.0; C n = *18*, 12.9 ± 4.5; NM n = *15*, 12.2 ± 4.4	1, **6** months							
								
	**Intervention:***Activities*: home–made toys were used for play sessions. *Time and frequency*: 1 h per day in hospital, 6 days per week; 1 h per week at the family home over 2 years after hospital treatment followed by 1 h per week over year 3 after hospital treatment. *Personnel*: nurse or health worker with limited training		*Griffiths Mental Scales DQ: *INT 96 ± 11.3, C 82 ± 12.1, NM 105 ± 11.2	*Griffiths Hearing & Speech: *INT 97 ± 17.6; C 74 ± 13.5; NM 93 ± 8.2	*Griffiths Locomotor: *INT 98 ± 17.4; C 90 ± 15.6; NM120 ± 20.5; *Hand & eye:* INT 101 ± 10.5; C 85 ± 14.6; NM 105 ± 13.0				
Grantham–McGregor 1983 [26]		18, **24** months	*Griffiths Practical Reasoning:* INT 93 ± 9.5; C 78 ± 12.1; NM 94 ± 14.3						
Grantham–McGregor 1987 [25]		36, 48, 60, **72** months	*Stanford–Binet Test:* INT 71 ± 8.3; C 64 ± 6.4; NM 76 ± 9.5	*PPVT:* INT 43 ± 4.6; C 39 ± 9.9; NM 47 ± 5.5		INT 92 ± 7.5; C 95 ± 8.4; NM 93 ± 6.8	INT 95 ± 2.5; C 94 ± 4.5; NM 101 ± 3.8		
Grantham–McGregor 1994 [24]		7, 8, 9, **14** years	*WISC full scale: *INT 65 ± 12.4; C 56 ± 9.4; NM 74 ± 12.7.	*PPVT:* INT 71 ± 11.9; C 64 ± 7.2; NM 77 ± 13.3.				INT 0.8 ± 0.6; C 1.0 ± 0.9; NM 0.3 ± 0.8	
			*WISC performance:* INT 64 ± 14.9; C 58 ± 12.5; NM 78.3 ± 11.2						
Nahar 2009 [28]	Quasi–experimental study, Bangladesh. Enrolment: 2002–2003; INT n = *33* 12.8 ± 4.7; C n = *37* 12.0 ± 4.6	**6** months	*BSID–II Mental raw score:* INT 103 ± 12.1; C 94 ± 8.8		*BSID–II Psychomotor raw score:* INT 67 ± 8.1; C 63 ± 8.2				INT 3.1 ± 0.9; C 3.6 ± 1.2
	**Intervention:**								
	*Activities*: children were taught about size, shapes, and numbers with visual aids mothers were shown feeding, bathing, and play activities during hospital stay; at home visits, toys were given and replaced at each visit. *Time and frequency:* daily 30 min group play sessions and daily 30 min individual sessions during 14 day hospital treatment; 11 sessions at family homes and 7 sessions at hospital within 6 months of discharge. *Personnel*: health workers with two weeks training.								

SD – standard deviation, INT – malnourished intervention group, C – malnourished control group, NM – non–malnourished intervention comparison group, DQ – development quotient, PPVT – Peabody Picture Vocabulary Test, WISC – Wechsler Intelligence Scale for Children, BSID–II – Bayley Scales of Infant Development, Second Edition

*Development and nutritional outcomes described in this table are from the latest follow–up times in the references (indicated in bold at the column ‘follow–up’). Some data was only presented in figure form in the different articles from Grantham–McGregor et al., and therefore could not be used in this table. The Grantham–McGregor 1989 paper is not included in table because this article presented only observational data.

### Evidence quality of included studies

**Figure 2:**
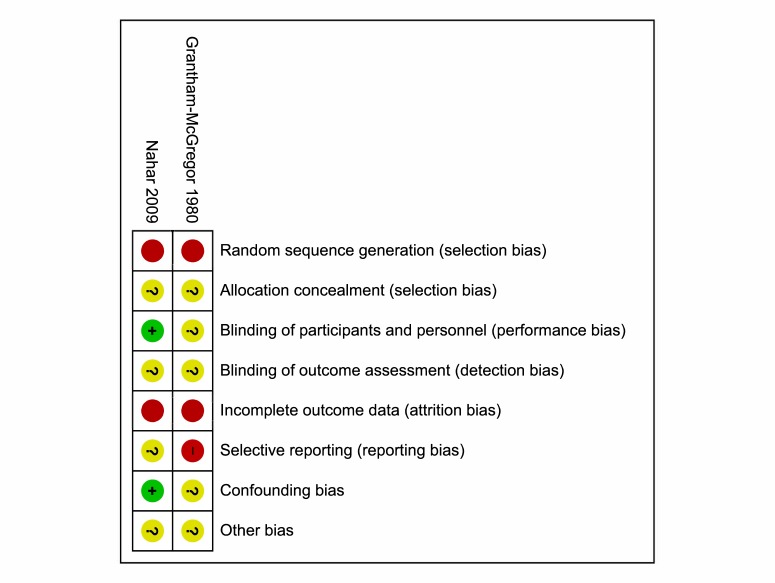
Risk of bias summary: review authors' judgements about each risk of bias item for each included study.

For the Grantham–McGregor 1980 study, there was high risk of selection bias (random sequence generation) and reporting bias at the study level, since all tests were conducted by a tester blind to the participants’ groups only from the 12–month session onwards; importantly the tester was not blinded at earlier assessment time points. There was also a high risk of attrition bias for all outcomes because of a lower number of children included in the non–intervention group in the 1987 publication than in the subsequent 1994 publication of this study. There was unclear risk for all other types of bias at the study and outcome levels (**Figure 2**).

There was a high risk of selection bias (random sequence generation) for the Nahar 2009 study as well due to the lack of randomization, in addition as high risk of attrition bias for all outcomes due to a high loss to follow–up. For all outcomes in the Nahar 2009 study, there was low risk of detection bias, as the tester was unaware of the participants’ groups, and confounding bias because covariates were specified and controlled for (**Figure 2**). The types of bias for both studies are listed in Table S4 and Table S5, respectively, in **Online Supplementary Document(Online Supplementary Document)**.

Overall, for each of the primary outcomes for this review and for mortality, evidence was of very low quality. Upgrading the quality of evidence because of a large magnitude of effect, confounding, or dose–response gradient was not admissible for any outcomes.

### Effects of psychosocial stimulation interventions

#### Child developmental outcomes

##### Cognitive development

Short–term cognitive outcomes (ie, at six months and two years after discharge from hospital) were significantly higher in children that received the intervention compared to the control group of malnourished children in the Grantham–McGregor 1980 study based on mean developmental quotients (DQ) from the Griffiths Mental Development Scales. The intervention group had the same DQ as the non–malnourished comparison group at two years’ follow–up. At five years’ follow–up, the Griffiths Mental Development Scales and the Stanford–Binet were both used to evaluate cognitive function. Results indicated that the intervention group had significantly higher DQ scores and intelligence quotients (IQ) compared with the control group, however the non–malnourished comparison group had the highest IQ scores (see **Table 2**). 14 years after leaving hospital, the Wechsler Intelligence Scale for Children was used to test the IQ of the participants. The intervention group had a significant higher IQ than the control group (mean IQ 65 ± 12.4 vs 56 ± 9.4, respectively), but lower than the non–malnourished comparison group (mean IQ 74 ± 12.7). In the Nahar 2009 study, the children in the intervention group had significantly higher mental raw scores of the Bayley Scales of Infant Development, Second Edition (BSID–II) than those of the children who did not receive the intervention at the six–month follow–up.

**Table 2:** Summary of findings table

Outcomes	Illustrative comparative risks* (95% CI)		Relative effect (95% CI)	No of Participants (studies)	Quality of the evidence (GRADE)†
Assumed risk (control)	Corresponding risk (psychosocial stimulation)
**Cognitive development (1):**					
Bayley Scales of Infant Development, Second Edition (Mental Development Index raw scores) and Griffiths Mental Development Index		The mean cognitive development in the intervention groups was **0.95 SD higher **(0.55 to 1.35 higher)		109 (2 studies)	⊕⊝⊝⊝, **very low**‡,§
Follow–up: 6 months (short–term)					
**Cognitive development (2):**					
Wechsler Intelligence Scale for Children	The mean cognitive development in the control groups was **56.1**	The mean cognitive development in the intervention groups was **8.6 higher** (1.3 to 15.9 higher)		35 (1 study)	⊕⊝⊝⊝, **very low** ‡,#
Follow–up: 14 years (long–term)					
**Language development:**					
Peabody Picture Vocabulary Test	The mean language development in the control groups was **63.9 (raw score)**	The mean language development in the intervention groups was **6.9 higher** (0.4 to 13.4 higher)		35 (1 study)	⊕⊝⊝⊝, **very low**‡,#
Follow–up: 14 years (long–term)					
**Motor development:**					
Bayley Scales of Infant Development, Second Edition (Psychomotor Development Index, raw scores) and Griffiths Mental Development Scales (locomotor subscale and eye and hand coordination subscale)	Not pooled	Not pooled	Not estimable	104 (2 studies)	⊕⊝⊝⊝, **very low**‡,§
Follow–up: 6 months (short–term)					
**Weight–for–length or weight–for–height:**	Not measured	Not measured	Not estimable		
**Length–for–age or height–for–age:**					
Z–scores. Scale from: –4 to 4	The mean length–for–age or height–for–age in the control groups was **–1.0 SD**	The mean length–for–age or height–for–age in the intervention groups was **0.2 higher** (0.3 lower to 0.7 higher)		35 (1 study)	⊕⊝⊝⊝, **very low** ‡,#¶
Follow–up: 14 years (long–term)					
**Weight–for–age:**					
Z–scores. Scale from: –4 to 4	The mean weight–for–age in the control groups was **–3.1 SD**	The mean weight–for–age in the intervention groups was **0.5 higher** (0.006 to 1.0 higher)		70 (1 study)	⊕⊝⊝⊝, **very low**¶,**
Follow–up: 6 months (short–term)					
**Mortality:**			RR 2.5 (0.5 to 12.3)	112 (2 studies)	⊕⊝⊝⊝, **very low**‡,#
Number of deaths					
Follow–up: 6 months to 14 years					

GRADE – Grading of Recommendations Assessment, Development and Evaluation, CI – confidence interval, RR – risk ratio

*The basis for the **assumed risk** (eg, the median control group risk across studies) is provided in footnotes. The **corresponding risk** (and its 95% confidence interval) is based on the assumed risk in the comparison group and the **relative effect** of the intervention (and its 95% CI).

**†**GRADE Working Group grades of evidence. **High quality**: Further research is very unlikely to change our confidence in the estimate of effect. **Moderate quality**: Further research is likely to have an important impact on our confidence in the estimate of effect and may change the estimate. **Low quality**: Further research is very likely to have an important impact on our confidence in the estimate of effect and is likely to change the estimate. **Very low quality**: We are very uncertain about the estimate.

‡Selection bias, attrition bias, and reporting bias are all likely.

§Clinical heterogeneity of outcome measures.

#Wide CI.

¶Z–scores according to the 1977 NCHS reference standards.

**Selection bias and attrition bias are likely.

Short–term academic performance was assessed in Grantham–McGregor 1980 study with Griffiths Mental Development; specifically, the performance subscale, indicating the speed of working an precision, and the practical reasoning subscale, describing the ability to solve problems, were used [30,31]. At two and five years’ follow–up, children in the intervention group scored in between the control group and the non–malnourished comparison group on the performance subscale. On the practical reasoning scale, children in the intervention group had similar scores to the non–malnourished children and had scores significantly ahead of the control group at two and five years’ follow–up. At 9 and 14 years’ follow–up, academic performance (ie, spelling and reading) was tested with the Wide Range Achievement Test; the intervention group scored intermediate between the control and non–malnourished comparison groups, although the difference in scores between the intervention and control groups was not significant.

**Table 2** shows short– and long–term cognitive development included as important outcomes. Since both studies reported on cognitive development at six months’ follow–up, results were pooled and the standardized mean difference was calculated. The standardized mean difference was 0.95 on cognitive development at six months between the two studies.

##### Language development

The intervention group in Grantham–McGregor 1980 study scored better than the control group at short–term language outcomes. They also scored significantly higher on the Griffiths Mental Development hearing and speech scale than the non–malnourished comparison group at two years’ follow–up. However, long–term follow–up scores on this scale were no longer significantly ahead of the control group.

Between three and six years’ follow–up in the Grantham–McGregor 1980 study, both the intervention and non–malnourished comparison groups had similar language scores on the Peabody Picture Vocabulary Test with significantly higher scores than the control group. At the 14–year follow–up, verbal performance was tested with the verbal scale of the Wechsler Intelligence Scale for Children; the intervention group scored significantly higher than the control group and had similar scores as the non–malnourished comparison group. Long–term language development results were included in **Table 2**.

##### Motor development

In the Grantham–McGregor 1980 study, motor development was tested with the locomotor subscale (ie, gross motor skills) and eye and hand coordination subscale (ie, fine motor skills) of the Griffiths Mental Development Scales [30,31]. At the two–year follow–up time, the intervention group had higher scores on gross motor skills compared to the control group, yet these scores were lower than the non–malnourished comparison group. At four years’ follow–up, the intervention and control groups scored similarly for gross motor skills.

In terms of fine motor skills, the intervention group in the Grantham–McGregor 1980 study had scores similar to those of the non–malnourished group at the two–year follow–up. The intervention group remained significantly ahead of the malnourished control group for fine motor skills at the three–, four–, and five–year follow–up times, and scored similarly to the non–malnourished children. In the Nahar 2009 study, motor development was assessed at the six–month follow–up time with the Psychomotor Developmental raw scores of the BSID–II. The intervention group had significantly better psychomotor raw scores than the control group. However, the difference in scores was lower than that of the mental raw score of the BSID–II, and the functional importance was not clear. Short–term motor development was described in **Table 2** yet results could not be pooled because of the differences in outcome measures, with the Nahar 2009 study only presenting psychomotor development raw scores including both fine and gross motor development. Since the Grantham–McGregor 1980 study used only figures to describe long–term motor development data, these results could not be included in **Table 2**.

#### Social–emotional outcomes

In the Grantham–McGregor 1980 study, behavior was assessed at the three–year follow–up time. The behavior of the mother and child was observed during a play situation. A non–standardized questionnaire was used for this observation. There was no significant difference identified between the intervention group and the non–malnourished comparison group. The control group of malnourished children stayed nearer to their mothers and stopped playing with their toys sooner. During the developmental assessments in the Nahar 2009 study, activity level, emotional tone, vocalization, and cooperation were observed with nine–point Likert scales adapted from Wolke et al [32]. There was no significant treatment effect identified in any of these behavior ratings in the Nahar 2009 study.

#### Child nutritional outcomes

In the Grantham–McGregor 1980 study, anthropometric measures including length– or height–for–age and weight–for–age, expressed as percentage of expected values for age and sex, were not significantly different between the groups six months and two years after hospital stay. There were also no significant differences in reported weight–for–height and height–for–age across the malnourished intervention and control groups at assessment time points between three and 14 years after hospital stay using percentage of expected values or z–scores based on the 1977 NCHS reference standards. Malnutrition relapse and readmission rates were not described.

In the Nahar 2009 study, duration of hospital stay was not significantly different between groups, indicating that inpatient nutritional recovery was similar. Weight–for–age z–scores (WAZ), WLZ, and length–for–age z–scores were reported at enrolment and at discharge. At the six–month follow–up time, only WAZ scores were reported. The mean difference in WAZ between the intervention group compared to the control group was clinically significant at follow–up, at a value of 0.4 SD higher in the intervention group (*P* = 0.029). No data on recurrence of malnutrition or readmission rates were reported. Long–term anthropometric outcomes are reported in **Table 2**, but for WAZ only short–term measures were done.

### Secondary outcomes

Mortality rates at the end of the 14 years of the Grantham–McGregor 1980 study were 14.2% in the intervention group, and there were no deaths in the control group (*P* = 0.11). It was reported that children in the intervention group died from accidents. In the Nahar 2009 study, mortality rates were 5.1% in the control group and 5.7% in the intervention group (*P* = 0.91); reasons were not specified. Other secondary outcomes, including quality of life and morbidities, were not described in either study.

## DISCUSSION

This systematic review contributes to the literature and demonstrates that the evidence supporting the WHO guidelines around provision of psychosocial stimulation during and after hospitalization is of very low quality across important outcomes in children with SAM. Neither of the included studies are randomized controlled trials, and there were high risks of different types of bias in both studies. Both studies examined hospital–based psychosocial intervention programs yet no studies that examined psychosocial stimulation interventions in children with SAM in the community were identified.

At the individual study level, each of the included studies showed significant differences between intervention and control groups of children with SAM in terms of child development. Cognitive development was significantly higher at short–term follow–up in the Nahar 2009 study and long–term follow–up in the Grantham–McGregor 1980 study in children with SAM who received psychosocial interventions. These children also had better language development at both short– and long term follow–up in the Grantham–McGregor 1980 study. These results are in line with a recent systematic review by Aboud & Yousafzai that evaluated psychosocial stimulation interventions in children under the age of two years who in low– and middle–income countries [5]. This recent review demonstrated a medium effect size of 0.42 and 0.47 on cognitive and language development, respectively [5]. However, at the 14–year follow–up period in the Grantham–McGregor 1980 study, both the malnourished intervention and control groups had poorer levels of academic performance compared to their non–malnourished peers, even after controlling for social background and hospitalization, possibly indicating long–term neurodevelopmental delays in children with SAM [24].

For motor development, there were mixed results between the two included studies. The psychosocial stimulation intervention did not have an effect on gross motor skills, but did improve fine motor skills in Grantham–McGregor 1980 study. The Nahar 2009 study also demonstrated significantly improved motor development scores, although the authors were not clear about whether or not this effect would be clinically significant. Both interventions used play activities and materials to stimulate fine motor development, which could explain the mixed outcomes for gross and fine motor development.

Both the Grantham–McGregor 1980 and Nahar 2009 studies used developmental assessment tools that were not culturally validated or locally standardized. Additionally, there is controversy about the validity of the Griffiths Mental Development Scales, which was used the most amount of times for the Grantham–McGregor 1980 study [33]. The BSID–II was used in the Nahar 2009 study; although it is a validated tool, it has since been replaced by the Bayley Scales of Infant Development, Third Edition [34]. Many children scored very low (ie, <50) on the BSID–II, and therefore the authors were not able to use standardized mental and psychomotor scores.

Nutritional outcomes did not change as a result of the psychosocial stimulation intervention in the Grantham–McGregor 1980 study. The Nahar 2009 study, on the other hand, did show statistically and clinically significant increases in WAZ scores in the intervention group compared to the control group six months after hospital stay. Authors hypothesized that psychosocial stimulation could improve mother–child interaction, which could lead to better feeding techniques [28]. Future research should evaluate the nutritional outcomes of psychosocial stimulation interventions in children with SAM and also explore possible mechanisms in more detail.

It is important to note that both studies included in this review applied interventions that differ from the current WHO recommendations of 15–30 minutes per day of psychosocial stimulation for children admitted to hospital with SAM [15]. Feasibility of the types of interventions tested in both included studies is of concern. For example, an intervention for three years after hospital stay, which was done in the Grantham–McGregor 1980 study, may not be feasible in most resource–constrained settings. During hospital stay, participants in the intervention group of the Grantham–McGregor 1980 study were involved in hour–long play sessions six days per week based on a semi–structured curriculum. This was followed by weekly home–based sessions for the first two years and bi–weekly sessions for an additional year. In the Nahar 2009 study, daily hour–long intervention sessions were done with participants during hospital stay, based on a child development manual with specific activities according to developmental milestones. There were also 18 follow–up visits with play activities as well as health and nutrition education (for hospital–based follow–up visits only), but after discharge there was a loss to follow–up of 39% of the children in the intervention group, vs 23% and 14%, respectively, in the control group.

Results from the two individual studies in this review showed important improvements in child development, indicating that further research is urgently needed to strengthen the case for psychosocial stimulation in children with SAM. Another important area to explore for improving child development outcomes in children with SAM is the added value of nutrition–specific interventions. Two recent systematic reviews in low– and middle–income countries in children under two years of age, not specifically in children with SAM, have found small benefits of nutritional interventions on child development outcomes, but the mechanisms explaining this relationship still need to be explored [5,35]. Additionally, the feasibility of psychosocial interventions should be investigated, especially since there is no data on how the basic WHO recommendations for play and stimulation activities for children with SAM in hospitals and health centers are currently practiced [12,15,36]. Compliance to psychosocial stimulation programs, and factors influencing their effectiveness such as maternal mental health, are also unknown. Last, in order to justify psychosocial interventions in areas with limited resources, the location (ie, hospital and/or community settings) and the optimal duration of psychosocial interventions should be a focus of further investigation with the use of reliable measures to understand if these interventions help to achieve the SDG for child development and other outcomes [8,12,37].

### Limitations

A limitation of this systematic review is that because there were few studies included and there was clinical heterogeneity of outcomes, it was not possible to conduct sub–group analyses and a meta–analysis.

To date, there are no randomized–controlled trials assessing psychosocial stimulation for children with SAM. Given that psychosocial interventions could be classified as behavioral interventions, it would be difficult to randomize individuals to groups and to conceal personnel at the study level. Authors of the Nahar 2009 study also explained that they also felt it would be unethical to provide the intervention to certain participants in the same hospital wards using randomization, and therefore used a time–lagged controlled design. This is one reason that the risk of bias is considered high and the quality low for the existing evidence. However, there are other possible strategies for randomization, such as block randomization of participant groups at frequent time intervals.

## CONCLUSIONS

Due to very low quality of evidence, there is currently insufficient direct evidence to recommend the provision of psychosocial stimulation in children with SAM. With SAM affecting millions of children worldwide, this is an important evidence gap. Results from the two individual studies in this review showed important improvements in child development in children with SAM, indicating that these interventions could be of benefit. More research is urgently needed to strengthen the case for psychosocial stimulation in children with SAM in the community and hospital settings. New studies also need to explore feasibility and implementation of psychosocial stimulation interventions in children with SAM.
