# Supplementary material for: Psychosocial stimulation interventions for children with severe acute malnutrition: a systematic review
Source: J Glob Health. 2017 Mar 30;7(1):010405. doi: 10.7189/jogh.07.010405 (PMC5441448; doi:10.7189/jogh.07.010405)
Supplement: Online Supplementary Document [file jogh-07-010405-s001.pdf]

Online Supplementary Document

Daniel et al. Psychosocial stimulation interventions for children with severe acute malnutrition: a systematic review

J Glob Health 2017;7:010405

Table S1. PRISMA 2009 checklist

Section/topic	#	Checklist item	Reported on page #
**TITLE**			
Title	1	Identify the report as a systematic review, meta-analysis, or both.	Title page
**ABSTRACT**			
Structured summary	2	Provide a structured summary including, as applicable: background; objectives; data sources; study eligibility criteria, participants, and interventions; study appraisal and synthesis methods; results; limitations; conclusions and implications of key findings; systematic review registration number.	1-2
**INTRODUCTION**			
Rationale	3	Describe the rationale for the review in the context of what is already known.	2-3
Objectives	4	Provide an explicit statement of questions being addressed with reference to participants, interventions, comparisons, outcomes, and study design (PICOS).	3
**METHODS**			
Protocol and registration	5	Indicate if a review protocol exists, if and where it can be accessed (e.g., Web address), and, if available, provide registration information including registration number.	1,4
Eligibility criteria	6	Specify study characteristics (e.g., PICOS, length of follow-up) and report characteristics (e.g., years considered, language, publication status) used as criteria for eligibility, giving rationale.	4-5
Information sources	7	Describe all information sources (e.g., databases with dates of coverage, contact with study authors to identify additional studies) in the search and date last searched.	4
Search	8	Present full electronic search strategy for at least one database, including any limits used, such that it could be repeated.	4, Supp. Appendix
Study selection	9	State the process for selecting studies (i.e., screening, eligibility, included in systematic review, and, if applicable, included in the meta-analysis).	6
Data collection process	10	Describe method of data extraction from reports (e.g., piloted forms, independently, in duplicate) and any processes for obtaining and confirming data from investigators.	6
Data items	11	List and define all variables for which data were sought (e.g., PICOS, funding sources) and any assumptions and simplifications made.	6

Risk of bias in individual studies	12	Describe methods used for assessing risk of bias of individual studies (including specification of whether this was done at the study or outcome level), and how this information is to be used in any data synthesis.	6
Summary measures	13	State the principal summary measures (e.g., risk ratio, difference in means).	7
Synthesis of results	14	Describe the methods of handling data and combining results of studies, if done, including measures of consistency (e.g., I^2) for each meta-analysis.	7

Section/topic	#	Checklist item	Reported on page #
Risk of bias across studies	15	Specify any assessment of risk of bias that may affect the cumulative evidence (e.g., publication bias, selective reporting within studies).	6-7
Additional analyses	16	Describe methods of additional analyses (e.g., sensitivity or subgroup analyses, meta-regression), if done, indicating which were pre-specified.	7
**RESULTS**			
Study selection	17	Give numbers of studies screened, assessed for eligibility, and included in the review, with reasons for exclusions at each stage, ideally with a flow diagram.	7-8, Tables and Figures, Supp. Appendix
Study characteristics	18	For each study, present characteristics for which data were extracted (e.g., study size, PICOS, follow-up period) and provide the citations.	8-9, Tables and Figures
Risk of bias within studies	19	Present data on risk of bias of each study and, if available, any outcome level assessment (see item 12).	9-10, Supp. Appendix
Results of individual studies	20	For all outcomes considered (benefits or harms), present, for each study: (a) simple summary data for each intervention group (b) effect estimates and confidence intervals, ideally with a forest plot.	10-15
Synthesis of results	21	Present results of each meta-analysis done, including confidence intervals and measures of consistency.	Tables and Figures
Risk of bias across studies	22	Present results of any assessment of risk of bias across studies (see Item 15).	9-10, Tables and Figures
Additional analysis	23	Give results of additional analyses, if done (e.g., sensitivity or subgroup analyses, meta-regression [see Item 16]).	-
**DISCUSSION**			
Summary of evidence	24	Summarize the main findings including the strength of evidence for each main outcome; consider their relevance to key groups (e.g., healthcare providers, users, and policy makers).	15-18
Limitations	25	Discuss limitations at study and outcome level (e.g., risk of bias), and at review-level (e.g., incomplete retrieval of identified research, reporting bias).	18-19
Conclusions	26	Provide a general interpretation of the results in the context of other evidence, and implications for future research.	19
**FUNDING**			

Funding	27	Describe sources of funding for the systematic review and other support (e.g., supply of data); role of funders for the systematic review.	Title page

From: Moher D, Liberati A, Tetzlaff J, Altman DG, The PRISMA Group (2009). Preferred Reporting Items for Systematic Reviews and Meta-Analyses: The PRISMA Statement. PLoS Med 6(7): e1000097. doi:10.1371/journal.pmed1000097

Table S2. Search strategy for MEDLINE.

**Subject string**	**Search details**
Severe acute malnutrition	(protein-energy malnutrition/ or severe acute malnutrition/ or kwashiorkor/ or wasting syndrome/ or hiv wasting syndrome/) or (severe* adj3 (malnutrition* or malnourish*)).tw.) or (kwashiorkor*.tw.) or (wasting adj (disease* or syndrome*)).tw.) or (marasmus.tw.)
Psychosocial stimulation or similar interventions	(Psychosocial Deprivation/) or (psychosocial*.tw.) or (psycho-social*.tw.) or (exp "Play and Playthings"/) or (Play Therapy/) or ((play or playing or plaything*).tw.) or (stimulation*.tw.) or ((responsive adj parent*).tw.)
Children	(infan* or newborn* or new-born* or neonat* or child* or adolescen* or juvenile or teen* or girl* or boy* or youth* or toddler* or paediatric* or pediatric*).mp. [***Age group Textword search terms***]

Table S3. Excluded studies with reasons for exclusion.

**Study**	**Reasons for exclusion**
Agarwal 1992	No psychosocial or similar intervention
Baubet 2003	Non-severely malnourished children included (p.611: children with mild, moderate, and severe malnutrition included);<br>No relevant child outcomes reported
Celedon 1980	Non-severely malnourished children included (p.29: some children over 60% of expected weight for age included)
Elizabeth 1997	Non-severely malnourished children included (p.682: children with moderate and severe malnutrition included)
El-khayat 2007	Non-severely malnourished children included (p.1774: children with WLZ up to -2 SD included)<br>No psychosocial or similar intervention
Goodfriend 2004	Narrative article rather than actual study
Hossain 2010	Non-severely malnourished children included (p.3: children with oedema or severe wasting excluded)
Lima 2008	No controls included
McLaren 1973	Non-severely malnourished children included (p.273: moderately undernourished children included)
Nahar 2012	Non-severely malnourished children included (p.702: children with oedema or severe wasting excluded)
Nahar 2015	Non-severely malnourished children included (p.485: children with oedema or severe wasting excluded)<br>No relevant child outcomes reported
Puentes-Rojas 1989	Non-severely malnourished children included (p.309: children with WLZ up to -1 SD included)

Table S4. Risk of bias table for the Grantham-McGregor 1980 study.

**Bias**	**Authors' judgement**	**Support for judgement**
Random sequence generation (selection bias)	High risk	Non-randomized controlled trial
Allocation concealment (selection bias)	Unclear risk	Insufficient information to permit judgement of 'Low risk' or 'High risk'
Blinding of participants and personnel (performance bias)	Unclear risk	Insufficient information to permit judgement of 'Low risk' or 'High risk'
Blinding of outcome assessment (detection bias)	Unclear risk	Only from the 12-month session onwards were tests conducted by a tester who was unaware of the subject's group
Incomplete outcome data (attrition bias)	High risk	Number of children included in the control group was lower in the 1987 publication than in the later 1994 publication; high risk of bias for all outcomes
Selective reporting (reporting bias)	High risk	Reporting of certain outcomes at various follow up times across the different publications for this study (e.g. anthropometric data)
Confounding bias	Unclear risk	Insufficient information to permit judgement of 'Low risk' or 'High risk'

Table S5. Risk of bias table for the Nahar 2009 study.

**Bias**	**Authors' judgement**	**Support for judgement**
Random sequence generation (selection bias)	High risk	Non-randomized controlled trial Quotation: "We conducted a time-lagged controlled study... A randomized trial was not possible..."
Allocation concealment (selection bias)	Unclear risk	Insufficient information to permit judgement of 'Low risk' or 'High risk'
Blinding of participants and personnel (performance bias)	Unclear risk	Insufficient information to permit judgement of 'Low risk' or 'High risk'
Blinding of outcome assessment (detection bias)	Low risk	Quotation: "A female tester, unaware of the children's group or study design, assessed the children..."
Incomplete outcome data (attrition bias)	High risk	High loss to follow-up in intervention group (i.e. 39% intervention vs. 14% control group lost, P=0.006); high risk of bias for all outcomes
Selective reporting (reporting bias)	Unclear risk	Insufficient information to permit judgement of 'Low risk' or 'High risk'
Confounding bias	Low risk	Covariates were specified and controlled for in the analysis; low risk of bias for all outcomes
